# Cost-effectiveness of olorofim in the treatment of invasive aspergillosis in patients with limited suitable alternative treatment options: a US payer perspective

**DOI:** 10.1128/aac.00570-25

**Published:** 2025-08-27

**Authors:** Thomas J. Walsh, Craig I. Coleman, Rob Blissett, Thibaud Prawitz, Giuseppe Bonetti, Magda Aguiar, Mark Bresnik, Belinda Lovelace

**Affiliations:** 1 Center for Innovative Therapeutics and Diagnostics, Richmond, Virginia, USA; 2 School of Pharmacy, University of Connecticut, Storrs, Connecticut, USA; 3 Maple Health Group 612467 , New York, New York, USA; 4 F2G Inc, Princeton, USA; University of Iowa, Iowa City, Iowa, USA

**Keywords:** antifungal therapy, olorofim, invasive fungal diseases, aspergillosis

## Abstract

This study aimed to estimate the cost-effectiveness of treating invasive aspergillosis (IA) in patients with limited treatment options with either olorofim or currently available antifungal salvage therapy from a US payer perspective. A hybrid decision tree-Markov model with a one-year time horizon was used to estimate health economic outcomes. The model considered probabilities of treatment response, mortality, and treatment-emergent adverse events; costs of antifungals and healthcare utilization; and patient utility. For olorofim, patient-level data from the open-label, single-arm, Phase IIb study of olorofim for the treatment of proven or probable IA with limited treatment options (Study 32, NCT03583164) was used. Salvage therapy data were based on an external control arm of a study of IA patients. A willingness-to-pay threshold of $50,000/quality-adjusted life-year (QALY) was assumed to assess the cost-effectiveness of olorofim versus salvage therapy. One-year costs (2023 USD) of treating IA in patients with limited alternative options were $208,696 for currently available salvage therapy and $167,971 for olorofim, for an incremental cost reduction of $40,725. QALYs were 0.46 for olorofim and 0.22 for salvage therapy. Olorofim was determined to be a dominant (less costly, more effective) strategy, with an incremental net monetary benefit of $52,827. Olorofim remained the dominant strategy across all sensitivity analyses. Upon probabilistic sensitivity analysis, olorofim was dominant in 90.0% of 1,000 iterations and cost-effective in 97.5%. Olorofim resulted in lower total treatment costs, antifungal costs, and more QALY gains versus currently available salvage therapy, making olorofim the dominant strategy for treating IA with limited treatment options.

## INTRODUCTION

Invasive fungal infections (IFIs) pose a substantial risk of illness and death, especially among individuals with weakened immune systems (1, 2). The frequency of IFIs is rising, driven by a global increase in the number of immunocompromised patients (1, 2).

Invasive aspergillosis (IA) is the most common fungal disease of the lungs. IA patients may develop cough, chest pain, and hemoptysis as the result of the fungus’s capacity to invade pulmonary blood vessels, resulting in ischemia and infarction of lung tissue (3, 4). The World Health Organization (WHO) designated *Aspergillus* as one of the four critical priority fungal pathogens, accounting for nearly 9,000 hospitalizations annually in the United States (US) and costing over $80,000/hospitalization (5, 6).

IA is principally treated with antifungal triazoles, including voriconazole, isavuconazole, and posaconazole (6, 7). The inpatient mortality in triazole-susceptible infections ranges between 19% and 20% (8). However, mortality for triazole-resistant *Aspergillus fumigatus*, which occurs in ~3% of patients (but can vary widely based upon underlying condition of patients and geographic region), is reported to range from 50% to 87% (9–12). Olorofim has been developed as a novel antifungal agent to fulfill a critical need for the treatment of triazole-resistant aspergillosis and for patients with limited therapeutic options (1, 13).

There is an urgent need for new classes of antifungal agents for IFIs caused by molds, including IA, with only three classes of antifungals currently Food and Drug Administration (FDA)-approved: polyenes, mold-active azoles, and echinocandins (1, 13). Importantly, antifungal resistance has emerged (9–12). Further limitations of existing classes include a limited number of oral therapy options, adverse events, and the potential for drug-drug interactions that can limit or complicate administration of chemotherapeutic or immunosuppressive agents (14).

Olorofim is an orally administered inhibitor of the class 2 fungal dihydroorotate dehydrogenase (DHODH) enzyme (1). An open-label, single-arm, Phase IIb study (Study 32, NCT03583164) evaluated olorofim’s efficacy and safety for treating IFI lacking suitable alternative treatment options, including IA patients with either proven or probable lower respiratory tract disease (15). We sought to compare the cost-effectiveness of treating proven or probable IA in patients with limited alternative treatment options with either olorofim or currently available salvage therapy.

## MATERIALS AND METHODS

We followed the 2022 Consolidated Health Economic Evaluation Reporting Standards (CHEERS) to report this cost-effectiveness analysis (16).

### Model structure and population

**Fig 1:**
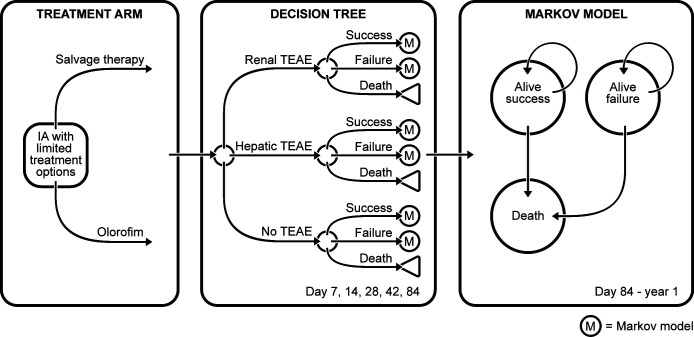
Diagram describing the hybrid structure of the model, where a decision tree with five time points (days 7, 14, 28, 42, and 84) is followed by a three-health-state (alive success, alive failure, and death) Markov model. IA = invasive aspergillosis; M = Markov node; TEAE = treatment-emergent adverse event.

We constructed a hybrid decision tree-Markov model in Microsoft Excel to evaluate the cost-effectiveness of treating patients with IA with limited alternative antifungal treatment options with either olorofim or currently available salvage therapy over a one-year time horizon (Fig. 1). Due to the length of treatment, setting, and available duration of follow-up, the model utilized a US healthcare payer perspective and only considered direct medical costs. No discounting of costs or outcomes was performed for the base-case analysis due to its one-year time frame.

The model assumed all patients were at least 18 years of age, with proven IFI caused by *Aspergillus* species or probable invasive pulmonary aspergillosis as defined by European Organization for Research and Treatment of Cancer (EORTC) and Mycoses Study Group (MSG) consensus definition (17). This model focused on salvage antifungal therapy among IA patients with limited treatment options and started at the time salvage therapy was deemed necessary. Patients started the decision tree in the “IA with limited treatment options” state, where they received either olorofim or currently available salvage therapy. Having limited treatment options was defined as ≥1 of the following in IA patients receiving one or more antifungal treatment regimens: known or predicted resistance of the infecting isolate to all licensed antifungal agents; failure to improve based on clinical and radiological assessment after at least 7 days of standard antifungal therapy; intolerance to available antifungal therapy; and inability to manage drug-drug interactions or failure to achieve therapeutic drug levels with current therapy, in combination with a contraindication to currently licensed antifungal therapies (6, 7). Currently available salvage therapy typically includes liposomal amphotericin B (L-AMB), echinocandins, or triazoles either alone or in combination (6, 7, 17–19). The structure of the decision tree reflected the timeframe of Study 32, where the model timepoints corresponded to each of the study assessment visits (days 7, 14, 28, 48, and 84) (15). The decision tree was followed by a three-state Markov model, where patients moved between the “alive success,” “alive failure,” and “death” health states over days 85–365 using a one-month cycle length.

### Treatment effects

For both olorofim and salvage therapy pathways, patients were first distributed based on treatment-specific probabilities of having a hepatic treatment-emergent adverse event (TEAE), a renal TEAE, or no TEAE, derived from Study 32 and prior literature (15, 20, 21). The model was then updated at each of the Study 32 follow-up time points (days 7, 14, 28, 42, and 84) with patients transitioning between the treatment success, treatment failure, and death health states. The probabilities of treatment success and failure (based upon Data Review Committee-adjudicated EORTC/MSG Clinical Response results for olorofim) or dying (an absorbing health state) were specific to olorofim (15) and salvage regimen assignment (19). For olorofim, patient-level data were used to derive probabilities for the 101 Study 32 patients with proven or probable IA who had limited or no alternative treatment options (15). For the currently available salvage therapy arm, probabilities were obtained from an external control arm of 86 IA patients requiring salvage therapy characterized by Walsh et al. (19). To obtain survival data from that study, the published Kaplan-Meier (KM) curve was digitized, and patient-level data were generated using the algorithm of Guyot and colleagues (22). The central point estimates from the KM curve were used to determine transition probabilities for death on salvage therapy at each timepoint. To incorporate the uncertainty around these point estimates into the sensitivity analyses, parametric survival models were fit to the survival data. A Gompertz distribution was used to generate the 95% confidence intervals around the hazard of survival (23). Clinical inputs are presented in Table 1.

**TABLE 1:** Clinical and economic inputs used in the model^*a*^

Parameter	Input	DSA		PSA	Source
		Lower value	Upper value	Distribution	
Length-of-stay coefficients (fraction of day per 24 hours spent in the health state)					
Ward – success	0.20	0.13	0.29	Log Normal	Maertens et al. (15)
Ward – failure	0.35	0.24	0.50	Log Normal	Maertens et al. (15)
Ward – death	0.74	0.45	1.00	Log Normal	Maertens et al. (15)
ICU – success	0.02	0.00	0.08	Log Normal	Maertens et al. (15)
ICU – failure	0.04	0.01	0.14	Log Normal	Maertens et al. (15)
ICU – death	0.10	0.02	0.64	Log Normal	Maertens et al. (15)
Total length of stay at day 84 (days)					
Ward – success	16.4	11.1	24.3	Calculation	Maertens et al. (15)
Ward – failure	29.1	20.3	41.6		
Ward – death	27.0	16.5	36.5		
ICU – success	1.6	0.4	6.8		
ICU – failure	3.2	0.8	11.8		
ICU – death	3.7	0.6	23.4		
Probability of treatment success – olorofim					
Day 84	33.7%	24.6%	43.8%	Beta	Maertens et al. (15)
Day 42	34.7%	25.5%	44.8%	Beta	Maertens et al. (15)
Day 28	40.6%	30.9%	50.8%	Beta	Maertens et al. (15)
Day 14	39.6%	30.0%	49.8%	Beta	Maertens et al. (15)
Day 7	28.7%	20.1%	38.6%	Beta	Maertens et al. (15)
Probability of treatment success – salvage therapy					
Day 84	25.6%	16.8%	36.1%	Beta	Walsh et al. (19)
Day 42	25.6%	16.8%	36.1%	Beta	Assumed same as day 84
Day 28	25.6%	16.8%	36.1%	Beta	Assumed same as day 84
Day 14	25.6%	16.8%	36.1%	Beta	Assumed same as day 84
Day 7	25.6%	16.8%	36.1%	Beta	Assumed same as day 84
Probability of death – olorofim					
Hazard death – olorofim	−5.62	−6.01	−5.24	Normal	Maertens et al. (15)
Day 84	25.7%	18.7%	36.0%	Beta	Maertens et al. (15)
Day 42	18.8%	9.8%	20.0%	Beta	Maertens et al. (15)
Day 28	10.9%	6.7%	13.8%	Beta	Maertens et al. (15)
Day 14	5.0%	3.4%	7.2%	Beta	Maertens et al. (15)
Day 7	4.0%	1.7%	3.7%	Beta	Maertens et al. (15)
Probability of death – salvage therapy					
Base case cumulative probability of death					
Day 84^*b*^	67.50%	NA^*d*^	NA	NA	Walsh et al. (19) (digitized from KM survival curve)
Day 42^*b*^	55.13%	NA	NA	NA	
Day 28^*b*^	51.00%	NA	NA	NA	
Day 14^*b*^	25.50%	NA	NA	NA	
Day 7^*b*^	12.75%	NA	NA	NA	
Gompertz death shape – salvage therapy	−0.02	−0.03	−0.01	Multivariate Normal	Gompertz distribution fitted to digitized data from Walsh et al. (19)
Day 84^*b*^	NA	52.23%	82.41%		
Day 42^*b*^	NA	44.80%	62.45%		
Day 28^*b*^	NA	38.08%	49.36%		
Day 14^*b*^	NA	25.55%	29.86%		
Day 7^*b*^	NA	15.20%	16.57%		
Gompertz death rate – salvage therapy	−3.63	−4.03	−3.23	Multivariate Normal	Gompertz distribution fitted to digitized data from Walsh et al. (19)
Day 84^*b*^	NA	51.54%	79.98%		
Day 42^*b*^	NA	39.66%	67.43%		
Day 28^*b*^	NA	31.68%	57.08%		
Day 14^*b*^	NA	19.49%	38.21%		
Day 7^*b*^	NA	10.94%	22.69%		
Probabilities used in the Markov model					
Probability of death (median survival)	3.5 years	2.37	5.16	Log Normal	Jansen et al. (24)
Probability of renal TEAE					
L-AMB	11.5%	7.6%	18.6%	Beta	Jansen et al. (24)
Salvage therapy	10.9%	7.0%	15.5%	Beta	Walsh et al. (19), Jansen et al. (24)
Additional ward days (on day 84) due to renal TEAE					
Without death	7.00	5.00	9.00	Normal	Ament et al. (20)
With death	3.04	1.85	4.23	Normal	Assumption
Probability of hepatic TEAE					
Olorofim	9.4%	6.1%	13.4%	Beta	Maartens et al. (15)
Azoles	3.6%	2.3%	5.1%	Beta	Herbrecht et al. (21)
Salvage therapy	1.8%	1.2%	2.6%	Beta	Walsh et al. (19), Herbrecht et al. (21)
Utility values					
IA with underlying disease state	0.59	0.53	0.65	Beta	Maertens et al. (15)
Treatment success	0.63	0.58	0.69	Beta	Maertens et al. (15)
Treatment failure	0.59	0.53	0.65	Beta	Maertens et al. (15)
IV therapy	−0.023	−0.039	−0.007	Normal	Matza et al. (25)
Cost inputs^*c*^					
Patient weight (kg)	70.00	42.56	97.44	Normal	Assumption
Drug acquisition costs per day (olorofim arm)					
Olorofim	$907.00	$586.9	$1,295.56	Gamma	Assumption
Drug acquisition costs per mg (AWP) (salvage therapy arm)					
L-AMB	$6.48	$4.19	$9.25	Gamma	Redbook
Echinocandin	$1.92	$1.24	$2.74	Gamma	Redbook
Mold-active triazole	$0.17	$0.11	$0.24	Gamma	Redbook
Resulting daily cost for the salvage therapy arm	$2,261.95	NA	NA	NA	Calculation
Health care resource utilization costs					
ICU stay (per day)	$6,408	$5,607	$8,010	Gamma	HCUPnet and Halpern et al. (26)
Ward stay (per day)	$3,204	$2,704	$3,704	Gamma	HCUPnet
IV antifungal at home (per visit)	$65	$23.11	$127.39	Gamma	CMS Physician Fee Schedules, CPT code 96365
Hepatic TEAE (cost for consult with specialist)	$135	$101	$198	Gamma	CMS Physician Fee Schedules, CPT code 99222
Antifungal makeup of the salvage therapy arm					
Antifungal Ttreatment Sstrategies				Percentage of salvage therapy	
L-AMB + mold-active triazole				45%	
L-AMB + echinocandin				40%	
L-AMB only				10%	
Mold-active triazole only				5%	
Echinocandin only				0%	

^*a*^ AWP = average wholesale price; DSA = deterministic sensitivity analysis; ICU = intensive care unit; IV = intravenous; kg = kilograms; L-AMB = liposomal amphotericin B; N = number; OR = oral; PSA = probabilistic sensitivity analysis; TEAE = treatment emergent adverse event.

^*b*^ Not varied in the DSA/PSA. The Gompertz estimates were varied instead, given that these estimates were correlated.

^*c*^ All costs are in 2023 USD.

^*d*^ NA, Not Applicable.

Patients who were still alive at the end of the decision tree entered the Markov model were assumed to stay either in the same treatment success/failure state (i.e., no transition between treatment success and treatment failure was considered) or die. In the Markov portion of the model, mortality was based on a median survival of 3.5 years similar to common underlying immunocompromising disease states (24).

During the decision tree, patients accrued days in the hospital general ward and intensive care unit (ICU) based on whether patients experienced treatment success, treatment failure, or death. Ward and ICU length-of-stay estimates for each outcome were derived from Study 32 (15) and were not treatment-specific. Analyses of the length-of-stay were conducted on Study 32 data using negative binomial regression. The coefficients were used to calculate the ward/ICU days progressively accrued over the decision tree timepoints (days 7, 14, 28, 42, and 84). For patients who died, accrual of ward/ICU days was assumed until death. For surviving patients, the accrual of ward/ICU days was assumed until the end of the time point when treatment response was reassessed.

### Costs

Throughout the decision tree model, costs captured were related to antifungal therapy, hospital ward or ICU lengths-of-stay, hepatic and renal TEAEs, and intravenous (IV) antifungal treatment at home (Table 1). The model assumed no difference in management of IA patients beyond day 84, and no costs were incurred during the Markov model.

Average wholesale prices (AWP) sourced from RedBook were used for antifungal drug costs (27). The estimated cost of olorofim was set to mirror a best estimate of the discounted cost of the comparator, L-AMB, in the ongoing Phase III clinical trial of olorofim for invasive aspergillosis (https://www.clinicaltrials.gov; NCT05101187). Currently available salvage therapy could include L-AMB, an echinocandin, or a mold-active triazole either alone or in combination. For echinocandins and triazoles, a conservative approach to estimate salvage therapy costs was used by assuming the lowest daily cost of any available agent in the class. The proportion of antifungal classes in the salvage therapy arm was informed by Walsh et al. (19) which provided rough proportions of different antifungals used (i.e., lipid or non-lipid formulations of amphotericin B, triazoles, combination amphotericin B and triazole, or other therapies (e.g., echinocandins) alone or in combination with amphotericin B or triazoles, and expert clinical opinion (consensus of three infectious disease and internal medicine clinicians from multiple institutions) to assign specific, contemporary salvage therapy choices (Table 1) (6, 7). Patients were assumed to remain on either antifungal treatment strategy for 84 days, consistent with the olorofim trial main treatment phase (15). Procurement and administration costs for patients receiving IV antifungals at home were sourced from the Centers for Medicare and Medicaid Services (CMS) Physician Fee Schedules, using the Healthcare Common Procedure Coding System (HCPCS) code 96365 (28). For those patients in the salvage therapy arm on IV antifungals, the costs for at-home treatment started after hospital discharge through day 84 or death.

Ward costs were estimated as average cost/day in hospital, sourced from National Inpatient Sample data for all hospitalizations using the Healthcare Cost and Utilization Project interface (HCUPnet) (29). The Russell equation (26) was used to estimate the cost/day in the ICU using an ICU-to-ward cost ratio of 2. Hospital length-of-stay costs were calculated as the product of ward and ICU costs/day and the durations of ward and ICU stays. The cost of a hepatic TEAE was that of a one-time gastroenterologist consultation (17). Patients experiencing a renal TEAE accrued seven additional ward days and associated costs (24). All costs used in the model were in 2023 US dollars.

### Health utility

Health utility values were assigned to treatment success and failure health states and applied throughout the entire model time horizon (Table 1). Health utility data were sourced from Study 32 (15), which collected data using the EuroQol-5 Dimension-5 Level (EQ-5D-5L) instrument at baseline, day 42, day 84, end of main treatment phase, and then during the extended study phase at baseline, every 4 weeks, and end of extended treatment. For patients who reported data across all five dimensions of the EQ-5D-5L and had both a baseline visit value and at least one post-baseline value, data were valued using the US tariff (30). Health utilities were modeled using mixed regression for repeated measurement (MRRM) to address the clustered nature of the health utility data. Following the results of the MRRM, the average health utility per health state of interest (treatment success or treatment failure) in the IA population was derived. The death health state was assigned a health utility value of zero. We assumed that for the first 7 days on treatment (i.e., before the first treatment response assessment and before any post-baseline utility assessment), patients had the same utility as the “treatment failure” state. Following day 7, patients were associated with a health utility value based on their achievement of treatment success at days 7, 14, 28, 42, and 84. The model assumed that patients on IV antifungal therapy had a health utility decrement of −0.023, applied only for the duration of the IV treatment (25).

### Model outputs

The model included the total treatment costs (2023 USD) for olorofim and currently available salvage therapy arms, as well as the cost breakdown per treatment arm (i.e., drug acquisition costs, healthcare resource utilization [HCRU], ward/ICU stays, hepatic and renal TEAEs and at-home antifungal IV administration). Health outcomes were measured in quality-adjusted life-years (QALYs). The cost-effectiveness results were presented in terms of the incremental cost-effectiveness ratio (ICER), which is the difference in costs divided by the difference in health outcomes. We expressed ICER as cost/QALY gained and incremental net monetary benefit (INMB), which represents the value of an intervention (olorofim) in monetary terms, calculated by multiplying the incremental benefits (QALYs) by the willingness-to-pay (WTP) threshold and then subtracting the incremental costs. A positive incremental INMB indicates that the intervention is cost-effective (or dominant) compared with the alternative at the given WTP threshold. The WTP threshold used in the base case analysis was $50,000/QALY.

### Sensitivity analyses

For the deterministic sensitivity analysis (DSA), each model parameter was assigned a point estimate and uncertainty parameters (i.e., “lower” and “upper” values). These values were set according to distributional information provided in the original source when available. If not available, the lower and upper values were assumed to be ±20% of the point estimate. The DSA varied one parameter at a time to assess the impact of each parameter independently on the model’s conclusions.

The probabilistic sensitivity analysis (PSA) drew values for each variable from their individual uncertainty distributions. For event rates and health utility values, a beta distribution was used to restrict draws to the range of 0 to 1. For costs and HCRU estimates, gamma distributions were fitted to prevent values less than zero.

Three scenario analyses were undertaken, including extending the time horizon to 5 years (costs and outcomes discounted at an annual rate of 3%); using a hospital perspective (analysis restricted to 28 days); and applying a hospital perspective while using alternative historical efficacy data for salvage therapy. For the latter scenario analysis, clinical response data from Walsh et al. (19) utilized combined data of the study’s external control arm and its prospective, experimental posaconazole arm.

A threshold sensitivity analysis was carried out to determine the maximum cost at which olorofim would remain cost-saving and cost-effective, assuming WTP thresholds ranging from $50,000/QALY to $300,000/QALY.

## RESULTS

### Base case analysis

The estimated one-year cost of treating proven or probable IA in patients with limited alternative antifungal options was $167,971 with olorofim and $208,696 with currently available salvage therapy, resulting in annual cost savings with olorofim of $40,725 (Table 2). Olorofim resulted in more QALYs gained (0.24 incremental QALYs) compared with currently available salvage therapy, making it the dominant treatment strategy (less costly and more effective). Assuming a WTP threshold of $50,000/QALY, the INMB was estimated to be $52,827, meaning that the cost to derive the benefit is less than the maximum amount that the decision-maker would be willing to pay for this benefit. The total one-year cost of the olorofim arm was lower than the currently available salvage therapy arm, with the main driver being the antifungal acquisition costs ($65,500 for olorofim versus $110,270 for salvage therapy) (Table 2). The olorofim arm resulted in modestly higher HCRU cost of $4,578.

**TABLE 2:** Results of the base case and scenario analyses^*a*^

Base case results					
Intervention	Total costs	Total LYs	Total QALYs	ICER	INMB
Salvage therapy	$208,696	0.370	0.220		
Olorofim	$167,971	0.764	0.462		
Incremental (deterministic)	−$40,725	0.393	0.242	Olorofim dominant	$52,827
Incremental (probabilistic)	N/A	N/A	N/A	Olorofim dominant	$52,553

Cost breakdown by treatment arm for the base case analysis					
		Salvage therapy	Olorofim		Incremental
Treatment costs		$110,270	$65,500		−$44,142
Healthcare resource use		$97,893	$102,471		$4,578
ICU		$19,018	$19,068		$50
Ward		$76,966	$83,390		$6,424
Renal TEAE		$1,907	$0		−$1,907
Hepatic TEAE		$2.40	$12.40		$10
IV antifungal at home		$532	$0		-$532

Scenario analyses results					
Scenario		Incremental QALYs gained	Incremental costs	ICER	INMB
5-year time horizon		0.787	−$40,725	Olorofim dominant	$80,082
Hospital perspective (28-days)		0.010	−$41,286	Olorofim dominant	$41,763
Hospital perspective (28-days) and alternative salvage therapy historic control		0.007	−$38,371	Olorofim dominant	$38,705

^*a*^ ICER = incremental cost-effectiveness ratio; INMB = incremental net monetary benefit; ICU = intensive care unit; IV = intravenous; LYs = life years; QALYs = quality-adjusted life years; TEAE = treatment emergent adverse event; NA = Not Applicable.

### Sensitivity and scenario analyses

**Fig 2:**
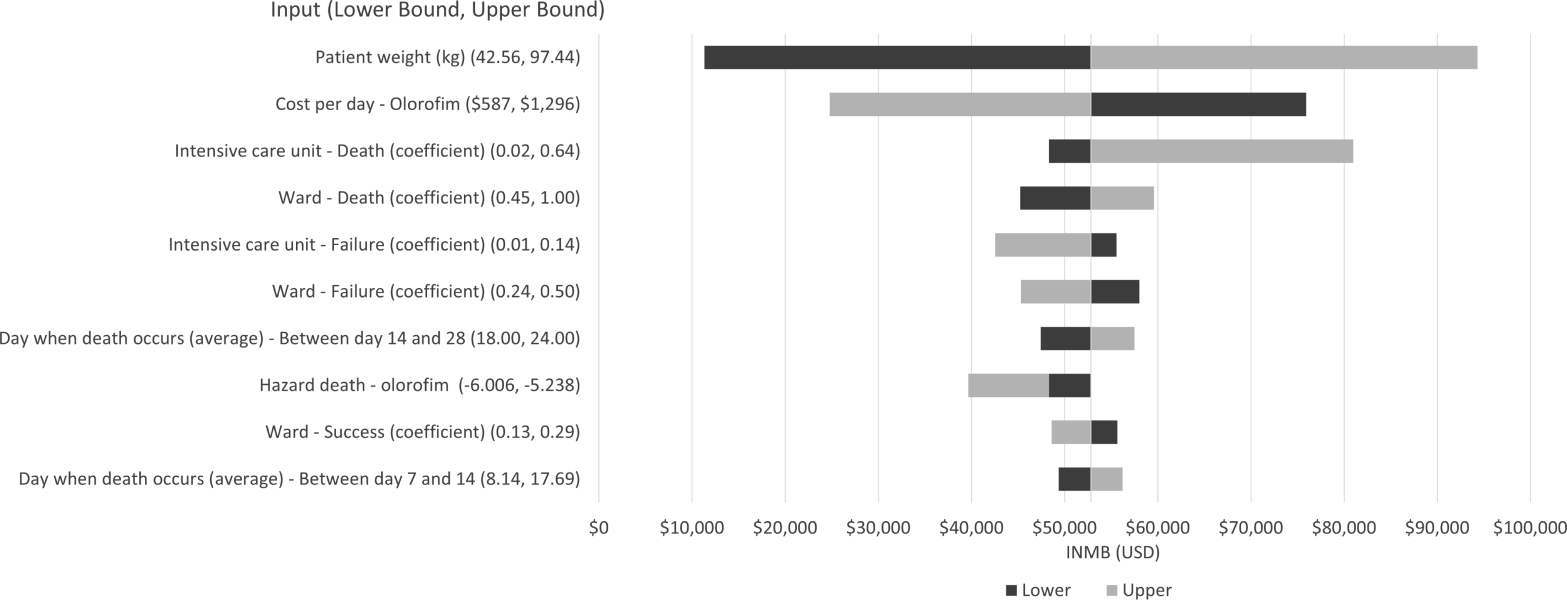
Deterministic sensitivity analysis (tornado plot).

The results of the DSA (one-way sensitivity analysis) suggested the inputs with greatest impact on the results were patient weight (as it influenced dosage and certain drug costs), cost/day of olorofim and ICU days accrued by the patients who die (Fig. 2).

**Fig 3:**
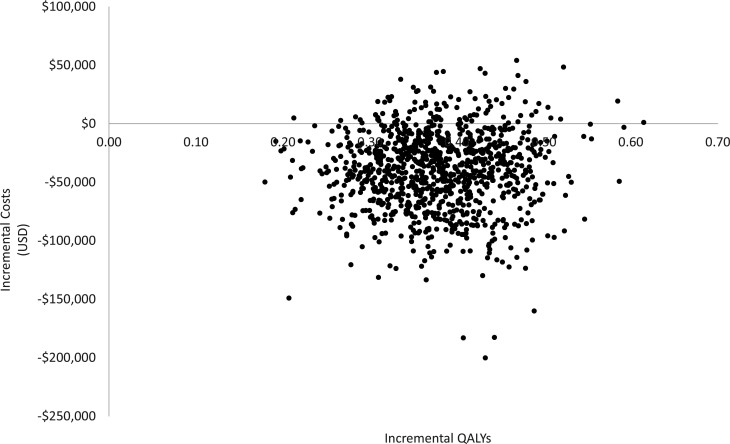
Probabilistic sensitivity analysis (cost-effectiveness plane). QALYs = quality-adjusted life years; USD = United States Dollars.

Results of the PSA in the form of a cost-effectiveness plane are presented in Fig. 3. Probabilistic results for INMB were consistent with the deterministic results, and at a WTP threshold of $50,000/QALY, olorofim was shown to be dominant in 90.0% and cost-effective in 97.5% of 1,000 iterations.

The model’s conclusions did not change in any of the scenario analyses, with olorofim being dominant (less costly, more effective) (Table 2).

**Fig 4:**
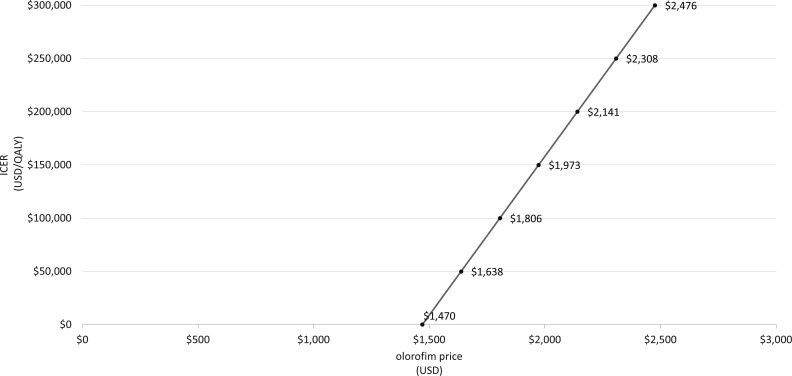
Threshold sensitivity analysis of olorofim cost. ICER = incremental cost-effectiveness ratio; QALY = quality adjusted life year; USD = United States Dollars.

The maximum cost at which olorofim would be cost neutral (ICER = $0/QALY gained) was $1,470/day. The acquisition cost at which olorofim was cost-effective ranged from $1,638/day at a WTP threshold of $50,000 to $2,476/day, at a WTP threshold of $300,000/QALY (Fig. 4).

## DISCUSSION

The estimated one-year cost savings was $40,725 and total incremental QALYs gained was 0.24 for olorofim compared with currently available salvage therapy. Thus, this economic model demonstrated olorofim to be the dominant treatment option compared to currently available salvage therapy in patients with IA with limited alternative treatment options.

The model results’ direction was maintained in both the DSA and PSA, showing its robustness in the face of parameter uncertainty. Among the most influential drivers of cost differences between the two cohorts was antifungal drug acquisition costs, with olorofim being $44,770 less costly than currently available salvage therapy over the model time horizon. Also, as olorofim is an oral medication, its use negates the need for outpatient IV administration, further reducing antifungal-related costs. As an added benefit of oral medication, the elimination of daily manipulation of percutaneously inserted and/or chronically indwelling central venous catheter may reduce the frequency of catheter-associated bacterial bloodstream infections, as well as the nursing time and materials for IV administered antifungal agents. For ambulatory patients, an oral olorofim formulation mitigates the inconvenience and cost of patient time needed to receive IV therapy. The lower total costs in the olorofim arm were achieved despite a modest ($4,578) increase in HCRU, driven by a better survival outcome modeled for olorofim with increased number of hospital ward days compared with currently available salvage therapy.

The cost of olorofim has yet to be established, and therefore, our value of $907/day is an assumption and intended to reflect a discounted cost of L-AMB (a frequent component of salvage therapy). Notably, we found olorofim would still be dominant versus salvage therapy up to an acquisition cost of $1,470/day and would remain cost-effective compared to salvage therapy if priced between $1,638 and $2,476/day, depending on the acceptable WTP threshold. IA occurs primarily in immunocompromised patients (2) and is relatively uncommon qualifying as a “rare disease” by the Orphan Drug Act definition (31). With rare disease status, the Institute for Clinical and Economic Review (32) suggests a WTP threshold up to $200,000/QALY as appropriate for an IA treatment such as olorofim.

There are few cost-effectiveness analyses of IA treatments in patients requiring salvage therapy. Herbrecht and colleagues performed a cost-effectiveness analysis of posaconazole versus a salvage therapy external control arm (33). Similar to our study, they found that patients treated with the newer antifungal agent (in this case posaconazole) had total drug costs over the entire duration of treatment that were lower than salvage therapy ($11,846 ± $12,406 versus $35,537 ± $73,059), and significantly greater survival compared with salvage therapy at every time point assessed (*P* = 0.0001). Unfortunately, they did not consider other key health economic outcomes, including HCRU or patient health utility.

Due to the urgent need for new salvage treatments for patients suffering from IA and the ethical challenges of randomizing such patients to placebo or already failed therapies, assessing the cost-effectiveness of olorofim based on Phase IIb trial data (Study 32) (15) without a randomized comparator arm, was justified. Due to the absence of individual patient-level data from Walsh et al. (19), no adjustment for possible imbalances in baseline characteristics between salvage IA populations in Study 32 (15) and Walsh et al. (19) was conducted. Nonetheless, a naïve comparison of these two studies indicates that the populations appear broadly similar in key aspects, including the proportion of patients enrolled due to failure or intolerance of prior antifungal therapy, those with pulmonary invasive aspergillosis, and those with moderate to high levels of immunosuppression. Moreover, the design features, as well as the inclusion and exclusion criteria between the study of Walsh et al. (19) and those of Study 32 (15), are consistent. Although Walsh et al. (19) was deemed the best available published data source for the salvage arm, what is currently considered the best salvage therapy may have changed since the study was conducted. The impact of such changes on our analysis is unclear.

Since the study period of 1996–2001 from which the control population was derived from Walsh et al. (19), there have been advances in supportive care in parallel with a wider range of immunosuppressive regimens, expanded use of mold-active antifungal prophylaxis, COVID-19-associated aspergillosis, influenza-associated aspergillosis, and the emergence of triazole-resistant *Aspergillus fumigatus* (6, 11, 34, 35). Each of these interventions and developments has contributed to a relatively wide variation in survival, hospital length-of-stay, supportive care resources, and adverse events. A review of representative clinical trials, prospective observational studies, and retrospective cohort studies of invasive aspergillosis of patients since 1997 reveals a survival rate that has varied from 21% to 81% (8, 36–42). Covariates contributing to this variability of survival include acuity of illness (41), transplantation (36, 42), triazole resistance (39), unresolved neutropenia (43), and need for salvage therapy (44). Length-of-stay for invasive aspergillosis also has varied during this time from a median of 15 to 39 days (41, 42). The all-cause mortality rate of 19% in Study 32 (15) is within the lower range of the representative studies, including the pivotal trial for primary treatment of IA with voriconazole versus amphotericin B (21,20,21) and more recent trials, such as the SECURE study (8). We therefore estimated that supportive care advances would be reflected in the mortality and response outcomes, and to some degree, resource utilization and adverse events. Given that healthcare resource use outcomes were not reported in the late 90s and early 2000s, we applied the hospital length-of-stay measures from Study 32 (olorofim) (15) to the comparator group (currently available salvage therapy) (19) as dictated by their treatment responses.

Following economic modeling best practices, simplifying assumptions were made to achieve a balance between the model’s complexity, the available data, and the overall suitability of the model to answer the research question (45). First, we did not assess the impact of ongoing costs after the initial 84-day treatment period in our model. This was because the economic model evaluated the “incremental” cost difference between olorofim and salvage therapy with currently available antifungals. If treatment costs (in both treatment cohorts) were allowed to accrue during the Markov phase of the model, any difference in cost observed would likely favor olorofim given that it was associated with lower treatment costs. Second, the model did not consider diagnostic imaging, serial biomarker measurement, drug wastage, antifungal treatment switching or re-treatment, therapeutic drug monitoring, drug-drug interactions, or dose adjustment. Importantly, therapeutic drug monitoring of azoles and dose adjustment of chemotherapy or immunosuppressants (common co-medications in IA patients) due to drug-drug interactions are more likely to occur with azoles (particularly, voriconazole and posaconazole) than olorofim due to their moderate-to-severe inhibition of the cytochrome P450 3A4 isoenzyme. If we had included these costs, this would have only grown the cost difference between olorofim and salvage therapy, making olorofim more cost-effective. Next, the rate of hepatic TEAEs was based upon data for voriconazole from the study by Herbrecht and colleagues (24) and was assumed to represent the risk for all azoles. While there is more recent data providing hepatic TEAEs for other azoles, they generally suggest there are similar rates of these TEAEs (46). Finally, it was assumed that TEAEs did not affect the patients’ quality of life and their health state occupancy.

### Conclusions

Olorofim is an economically dominant alternative to currently available salvage therapy for IA patients with limited suitable alternative treatment options from a US payer perspective. At its estimated price, olorofim resulted in lower total one-year treatment costs and antifungal therapy costs, and more QALYs gained. With the unmet need for novel antifungals to treat rare IFIs such as IA, new cost-effective treatments would fill a gap in care. This model will inform early clinical and payer decision-making for olorofim-eligible IA patients with limited suitable alternative treatment options.
